# Proteomic dataset of the sea urchin Paracentrotus lividus adhesive organs and secreted adhesive

**DOI:** 10.1016/j.dib.2016.04.002

**Published:** 2016-04-22

**Authors:** Nicolas Lebesgue, Gonçalo da Costa, Raquel Mesquita Ribeiro, Cristina Ribeiro-Silva, Gabriel G. Martins, Valeria Matranga, Arjen Scholten, Carlos Cordeiro, Albert J.R. Heck, Romana Santos

**Affiliations:** a Netherlands Proteomics Center, Padualaan 8, 3584 CH Utrecht, The Netherlands; b Biomolecular Mass Spectrometry and Proteomics, Bijvoet Center for Biomolecular Research and Utrecht Institute of Pharmaceutical Sciences, Utrecht University, Padualaan 8, 3584 CH Utrecht, The Netherlands; c Centro de Química e Bioquímica, Departamento de Química e Bioquímica, Faculdade de Ciências da Universidade de Lisboa, Campo Grande, 1749-016 Lisboa, Portugal; d Departamento de Química e Bioquímica, Faculdade de Ciências, Universidade de Lisboa, 1749-016 Lisboa, Portugal; e Laboratório de FTICR e espectrometria de massa estrutural, Faculdade de Ciências, Universidade de Lisboa, 1749-016 Lisboa, Portugal; f Instituto Gulbenkian de Ciência, R. da Quinta Grande 6, 2780-156 Oeiras, Portugal; g Centro de Ecologia, Evolução e Alterações Ambientais, Faculdade de Ciências da Universidade de Lisboa, Campo Grande, 1749-016 Lisboa, Portugal; h Consiglio Nazionale delle Ricerche, Istituto di Biomedicina e Immunologia Molecolare, ‘Alberto Monroy’, Via Ugo La Malfa 153, 90146 Palermo, Italy; i MARE – Centro de Ciências do Mar e do Ambiente, Faculdade de Ciências da Universidade de Lisboa, Campo Grande, 1749-016 Lisboa, Portugal

**Keywords:** Sea urchin, Paracentrotus lividus, Tube feet, Secreted, Adhesive, Quantitative proteomics

## Abstract

Sea urchins have specialized adhesive organs called tube feet, which mediate strong but reversible adhesion. Tube feet are composed by a disc, producing adhesive and de-adhesive secretions for substratum attachment, and a stem for movement. After detachment the secreted adhesive remains bound to the substratum as a footprint. Recently, a label-free quantitative proteomic approach coupled with the latest mass-spectrometry technology was used to analyze the differential proteome of *Paracentrotus lividus* adhesive organ, comparing protein expression levels in the tube feet adhesive part (the disc) versus the non-adhesive part (the stem), and also to profile the proteome of the secreted adhesive (glue). This data article contains complementary figures and results related to the research article “Deciphering the molecular mechanisms underlying sea urchin reversible adhesion: a quantitative proteomics approach” (Lebesgue et al., 2016) [1]. Here we provide a dataset of 1384 non-redundant proteins, their fragmented peptides and expression levels, resultant from the analysis of the tube feet differential proteome. Of these, 163 highly over-expressed tube feet disc proteins (>3-fold), likely representing the most relevant proteins for sea urchin reversible adhesion, were further annotated in order to determine the potential functions. In addition, we provide a dataset of 611 non-redundant proteins identified in the secreted adhesive proteome, as well as their functional annotation and grouping in 5 major protein groups related with adhesive exocytosis, and microbial protection. This list was further analyzed to identify the most abundant protein groups and pinpoint putative adhesive proteins, such as *Nectin*, the most abundant adhesive protein in sea urchin glue. The obtained data uncover the key proteins involved in sea urchins reversible adhesion, representing a step forward to the development of new wet-effective bio-inspired adhesives.

**Specifications Table**

**Table:** 

Subject area	Biology
More specific subject area	Proteomics analysis of sea urchin adhesive organs and secreted adhesives.
Type of data	Figure, table
How data was acquired	Mass spectrometry, LC-MS/MS using a Orbitrap Q-exactive mass spectrometer (Thermo Scientific).
Data format	Raw, processed/analyzed
Experimental factors	Dissected tube feet to separate discs from stems, and collected adhesive footprints.
Experimental features	To perform the differential proteome of the sea urchin *Paracentrotus lividus* adhesive organs, tube feet were dissected to separate discs (adhesive part) from stems (non-adhesive part). Then both samples were in-solution digested with Lys-C and trypsin and. The resulting peptides were pre-fractionated by Strong Cation Exchange chromatography and the resulting fractions analyzed by LC-MS/MS, followed by database searching (UniProt/SwissProt for SEA_URCHINS) using the Mascot search algorithm. Adhesive footprints were also collected, followed by protein purification by SDS-PAGE and in-gel digestion of the obtained concentrated protein bands with the above-mentioned proteases. The obtained peptides were analyzed by LC-MS/MS and database searching.
Data source location	Netherlands Proteomics Center, Utrecht, Netherlands
Data accessibility	Data is within this article and have been deposited to the ProteomeXchange Consortium [2] via the PRIDE partner repository with the dataset identifier PRIDE: PXD003122.

**Value of the data**

• The expression levels of 1384 sea urchin tube feet proteins as well as their functional annotation can be useful to compare with lists of proteins identified in other adhesive organs.
• The methodology used to obtain the differential proteome of an adhesive organ that allowed us to reveal 163 key proteins specifically involved in sea urchin reversible adhesion can be of interest to be applied by other laboratories focusing on other attaching organisms and/or types of biological adhesion.
• Data present also a list of 611 annotated proteins present in the adhesive secreted by sea urchins and can be useful for laboratories working on the identification of biological adhesives.
• Present data can be further explored in other to find key functionalities in sea urchin adhesive proteins and by comparison with other marine adhesive proteins, significantly simplify the development of bio-inspired adhesives.

## 1 Data

This dataset provides a comprehensive list of non-redundant proteins, their fragmented peptides and expression levels, resultant from the analysis of the tube feet differential proteome. In addition, the comparison of protein expression levels in the adhesive (disc) versus the non-adhesive (stem) part of sea urchin tube feet, allowed the selection of highly over-expressed tube feet disc proteins (>3-fold), likely representing the most relevant proteins for sea urchin reversible adhesion, which were further annotated in order to determine the potential functions. In addition, we provide a list of non-redundant annotated proteins identified in the sea urchin secreted adhesive (glue). This list was further analyzed to identify the most abundant protein groups and pinpoint putative adhesive proteins.

## 2 Experimental design, materials and methods

### 2.1 Sample preparation

Sea urchins from the species *Paracentrotus lividus* (Lamarck 1816) were collected at low tide on the west coast of Portugal (Estoril, Portugal). Tube feet and the secreted glue for proteomics analysis were collected as elsewhere described [1].

### 2.2 Protein extraction, digestion and fractionation by strong cation exchange (SCX)

**Fig. 1:**
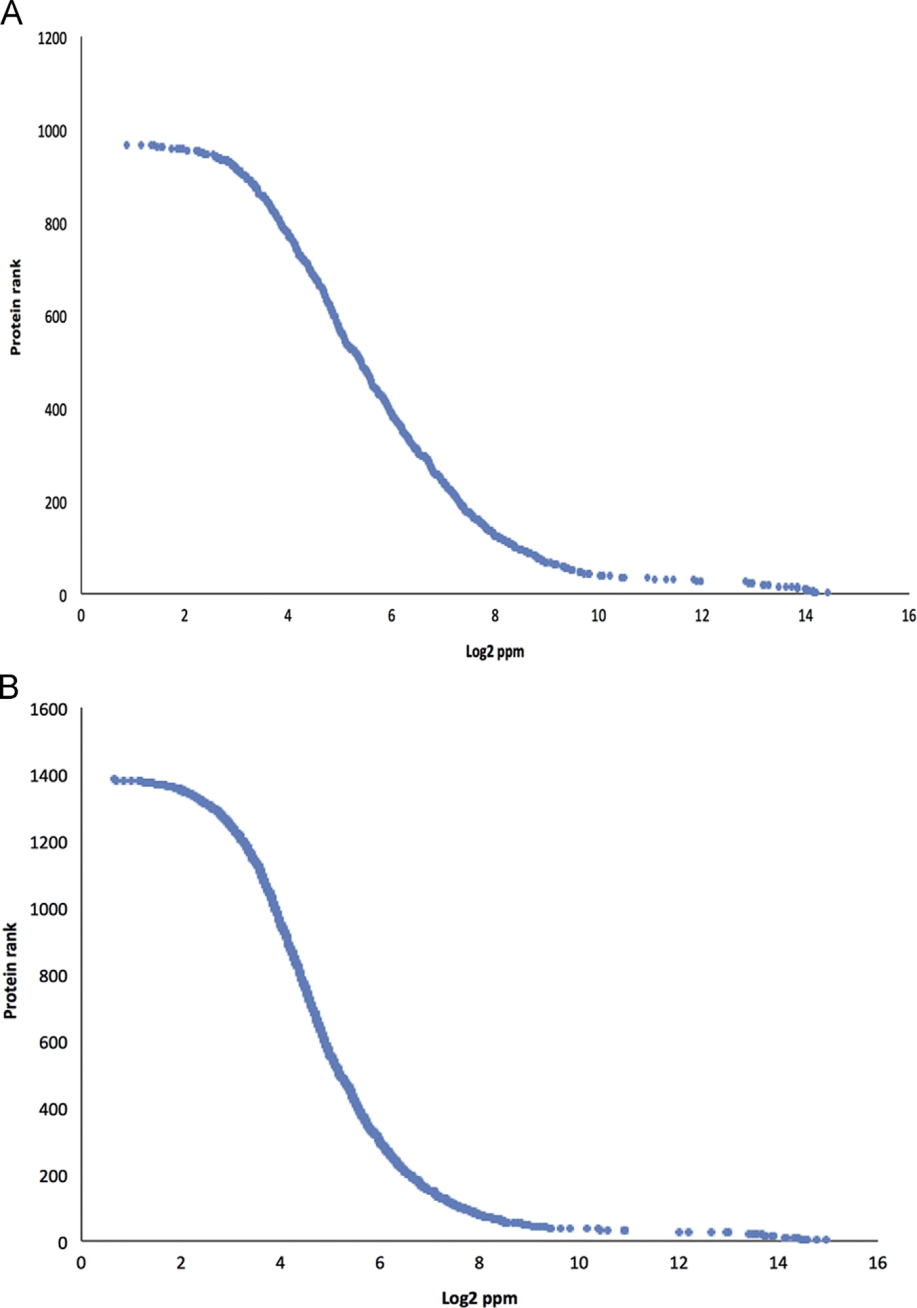
Paracentrotus lividus tube feet disc (A) and stem (B) in-solution digest fractionation gradient by strong cation exchange.

After collection, tube feet (disc and stem) and glue proteins were extracted and subsequently digested with Lys-C and trypsin as described in [1]. The digests from tube feet disks and stems were fractioned using SCX prior to mass spectrometry analysis [1]. A total of 50 SCX fractions (Fig. 1) were collected and the 25 most peptide-rich fractions used for subsequent LC–MS/MS analysis.

### 2.3 Analysis by liquid chromatography coupled to mass spectrometry

**Fig. 2:**
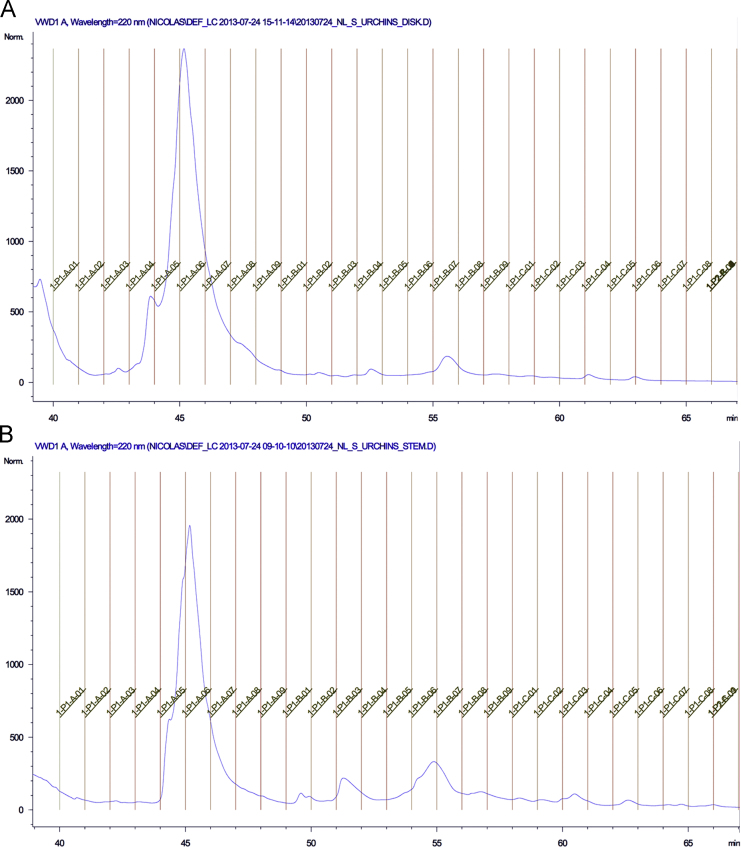
Paracentrotus lividus tube feet disc (A) and stem (B) proteins dynamic range.

Nanoscale liquid chromatography coupled to tandem mass spectrometry (LC–MS/MS) was performed for each SCX fractions on a reversed-phase easy nano-LC 1000 (Thermo Fisher Scientific, Odense, Denmark) coupled to an Orbitrap Q-exactive mass spectrometer (Thermo Scientific, Bremen, Germany) using higher-energy collisional dissociation (HCD) fragmentation [1]. RAW output files were submitted to Mascot (version 2.5.1) via Proteome Discoverer (version 1.3, Thermo Fisher Scientific) and searched against UniProt/SwissProt database for sea urchin (taxonomic Identifier: 7656) joined with the protein sequence encoded by the recently discovered Nectin mRNA variant [2] (GenBank KT351732) which was manually added. The mass spectrometry proteomics raw data as well as search results have been deposited to the ProteomeXchange Consortium [3] via the PRIDE partner repository with the dataset identifier PRIDE: PXD003122. The obtained protein dynamic range is presented in Fig. 2 and the list of identified proteins is presented in Supplementary file S1.

### 2.4 Data analysis

**Fig. 3:**
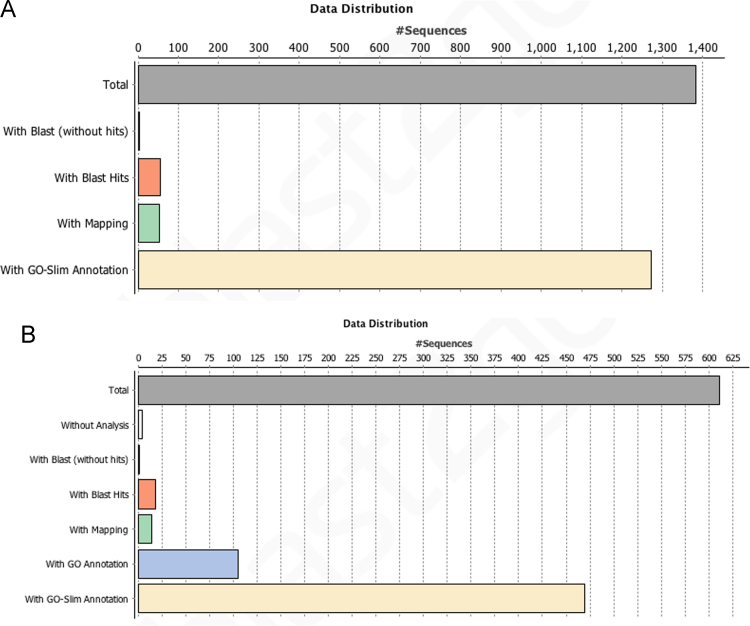
Annotation distribution of the proteins identified in *Paracentrotus lividus* tube feet (A) and the proteins identified in secreted adhesive (B).

Tube feet disc and stem differential proteome PSMs were normalized using the cytoplasmic actin of *Lytechinus variegatus* (O18548; Σ coverage: 85,19%) as a reference protein, while the adhesive proteome PSMs were normalized using the MW of each identified protein. Since a high percentage of the identified proteins were uncharacterized, Blast2GO 3.0.7 was used for functional analysis of the identified proteins [4]. The annotation output for the tube feet differential proteome and the secreted adhesive proteome are presented in Fig. 3.

**Fig. 4:**
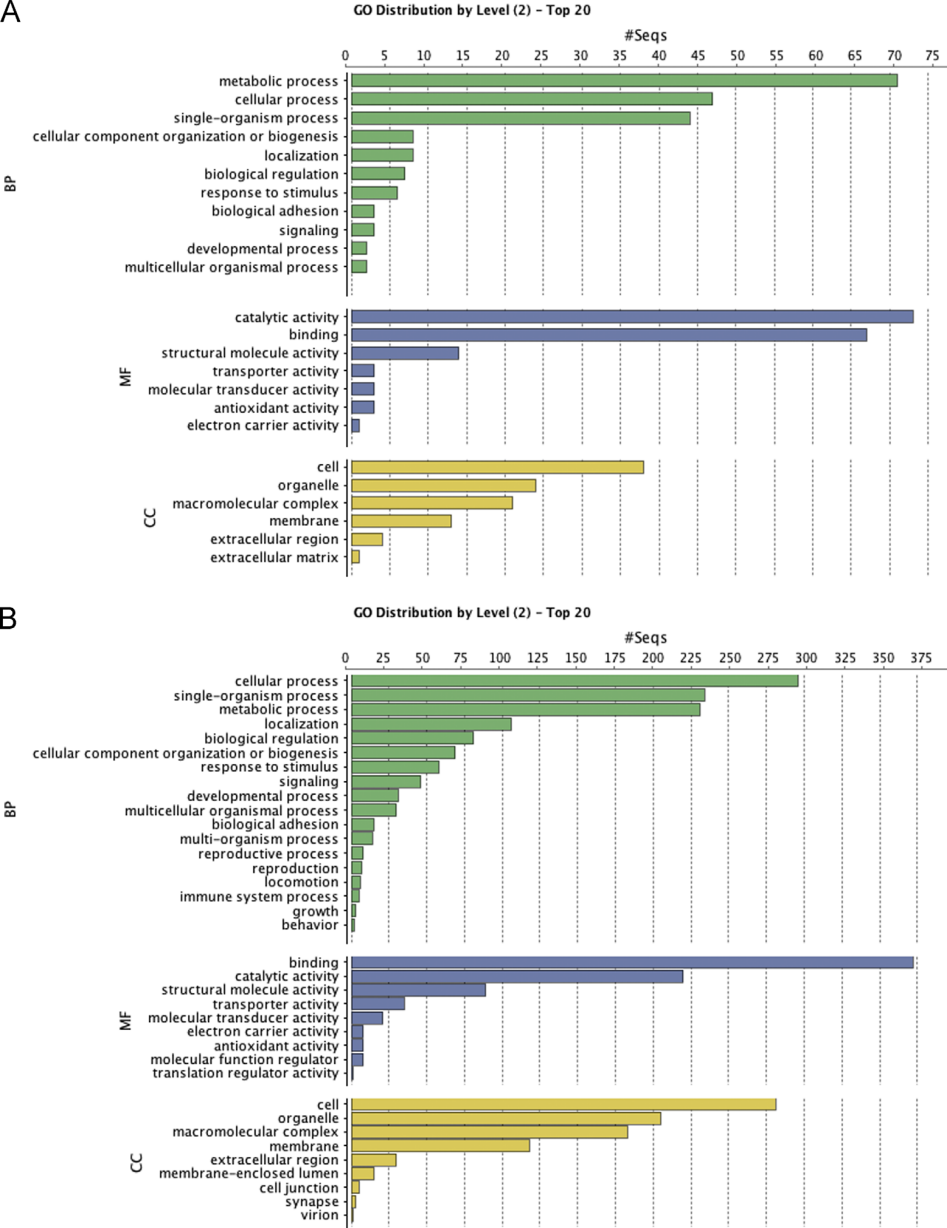
Gene Ontology distribution of the proteins identified in *Paracentrotus lividus* tube feet (A) and the proteins identified in secreted adhesive (B), grouped by biological process (BP), molecular function (MF) and cellular component (CC).

To group all identified proteins in selected subgroups of GO categories (biological process, molecular function and cellular compartment) the analysis tool of combined graph was used using a sequence filter of 20 for a more compacted view of the data. The GO protein distribution for the tube feet differential proteome and the secreted adhesive proteome are presented in Fig. 4.

## 3 Multiple sequence alignment

**Fig. 5:**
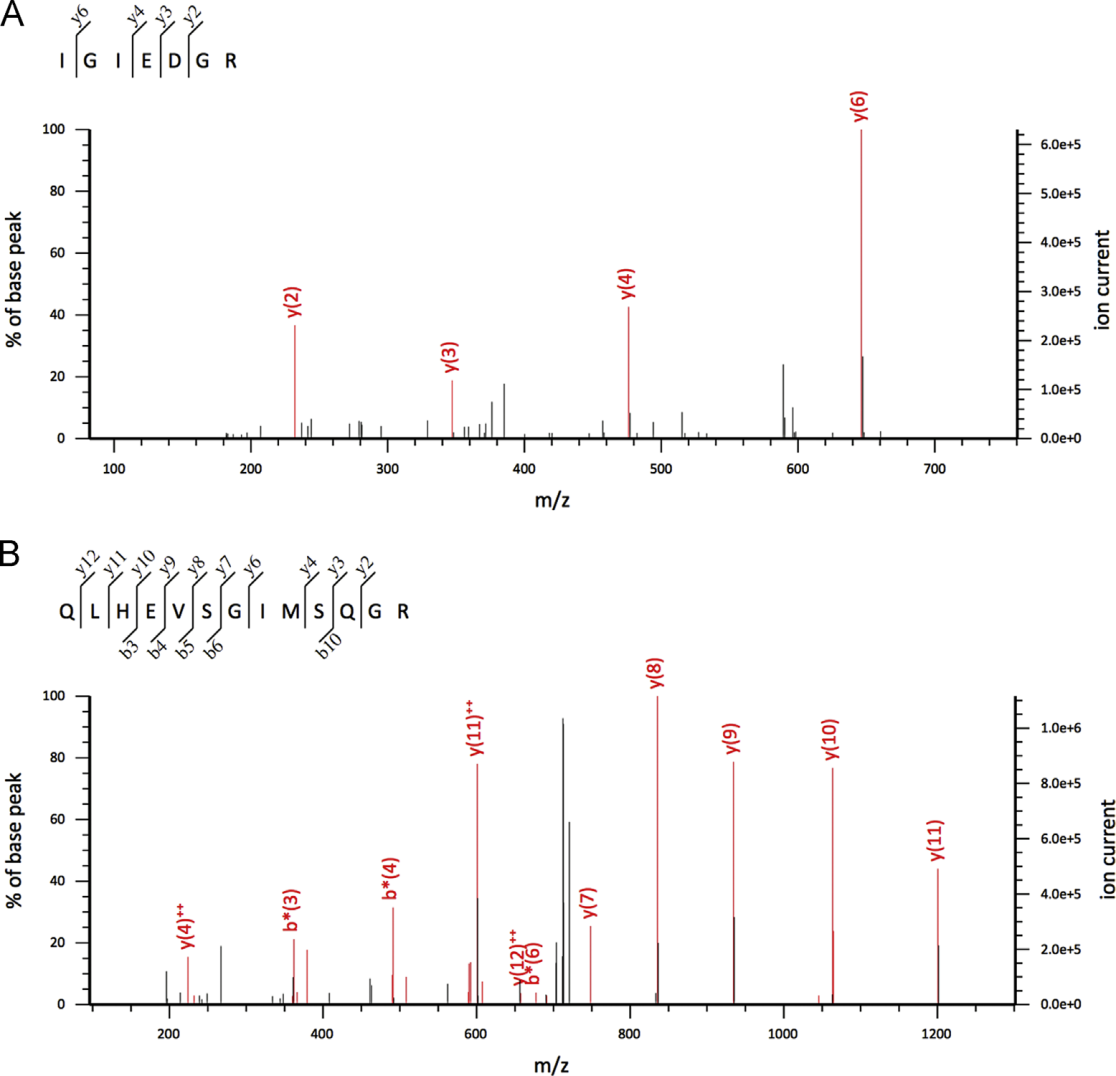
MS/MS spectra for the unique peptide IGIEDGR from the overexpressed *Nectin*-like variant W4Z4Y0 (A) and the unique peptide unique peptide QLHEVSGImSQGR from the overexpressed *Nectin*-like variant W4ZF96.

CLUSTAL 2.1 software was used to perform multiple sequence alignments of the recently identified *Nectin*-like protein variants (Q70JA0, KT351732, W4ZF96, W4Z4Y0) [1]. A comparison of the identified *Nectin* variants is presented in Supplementary file S2. Two of these new putative *Nectin* variants (W4ZF96, W4Z4Y0) were identified both in tube feet disks and the secreted adhesive, but based on one unique peptide each (Fig. 5), although one of the variants (W4Z4Y0) presented two more peptides in common with the previously know *Nectin* variants (Q70JA0, KT351732).

## Appendix A Supplementary material

Supplementary material

Supplementary material: **Supplementary file S1**. Comprehensive lists of non-redundant proteins, fragmented peptides, expression levels and functional annotation, resultant from the analysis of the tube feet differential proteome and the secreted adhesive proteome.

Supplementary material: **Supplementary file S2**. Multiple sequence alignment of the Nectin variants identified in Paracentrotus lividus tube foot differential proteome and in the secreted adhesive material proteome. Identic amino acids among the different variants relatively to Nectin Q70JA0 are in green, peptides identified by MS/MS are in bold, repetitive domains are highlighted in yellow.

## References

[1] Lebesgue N., da Costa G., Ribeiro R.M., Ribeiro-Silva C., Martins G.G., Matranga V., Scholten A., Cordeiro C., Heck A.J.R., Santos R. (2016). Deciphering the molecular mechanisms underlying sea urchin reversible adhesion: a quantitative proteomics approach. J. Proteom..

[2] Toubarro D., Gouveia A., Ribeiro R.M., Simões N., da Costa G., Cordeiro C., Santos R. (2016). Cloning, characterization and expression levels of the Nectin gene from the tube feet of the sea urchin Paracentrotus lividus. Mar. Biotechnol..

[3] Vizcaíno J.A., Deutsch E.W., Wang R., Csordas A., Reisinger F., Ríos D., Dianes J.A., Sun Z., Farrah T., Bandeira N., Binz P.A., Xenarios I., Eisenacher M., Mayer G., Gatto L., Campos A., Chalkley R.J., Kraus H.J., Albar J.P., Martinez-Bartolomé S., Apweiler R., Omenn G.S., Martens L., Jones A.R., Hermjakob H. (2014). ProteomeXchange provides globally co-ordinated proteomics data submission and dissemination. Nat. Biotechnol..

[4] Conesa A., Götz S., Garcia-Gomez J.-M., Terol J., Talon M., Robles M. (2005). Blast2GO: a universal tool for annotation, visualization and analysis in functional genomics research. Bioinformatics.

