# Supplementary material for: Proteomic dataset of the sea urchin Paracentrotus lividus adhesive organs and secreted adhesive
Source: Data Brief. 2016 Apr 22;7:1497–505. doi: 10.1016/j.dib.2016.04.002 (PMC4857396; doi:10.1016/j.dib.2016.04.002)
Supplement: Supplementary file 3 — Supplementary material: Supplementary file S2. Multiple sequence alignment of the Nectin variants identified in Paracentrotus lividus tube foot differential proteome and in the secreted adhesive material proteome. Identic amino acids among the different variants relatively to Nectin Q70JA0 are in green, peptides identified by MS/MS are in bold, repetitive domains are highlighted in yellow. [file mmc3.pdf]

CLUSTAL 2.1 Multiple Sequence Alignments

Accession	Length	Accession	Length	Score
Q70JA0	983	KT351732	984	98.47
		W4ZF96	387	73.64
		W4Z4Y0	649	57.32

```
                                       SIGNAL PEPTIDE             1st FA58C
Q70JA0      -----------------MAISHNALMVVLIVKMMIATSLQAQLGMEDGRLTDVQITASSC 43
KT351732    -----------------MAISHNALMVVLIVKMMIATSLQAQLGMEDGRLTDVQITASSC 43
W4ZF96      -----------------MATAK-VALVLLLVKMMIASSAQEQLGMQDGRITDAQITASSC 42
W4Z4Y0      MLPLLVAARYVRILPQTWNNRISLRFELLGIRPTNVSSVPGKIGIEDGRVDTTSISASSC 60

Q70JA0      FDGNHCTDRARLNQPLGNGKTGAWSARTNDQDQYIQVDLRQLHEVSGVMTQGRNG--YSQ 101
KT351732    FDGNHCTDRARLNQPLGNGKTGAWSARTNDQDQYIQVDLRQLHEVSGVMTQGRNG--YSQ 101
W4ZF96      YDQNHCTDRARLNQPRTSSKTGAWSARTNTPDQFIQADLRQLHEVSGIMSQGRNG--YTQ 100
W4Z4Y0      HDNNHCVARSRLNQPQEGVLMGAWSASTNDVNQWIQVDLRAAFEITGVITQGRKSSIFRQ 120

Q70JA0      WVTSFRVLSSLDGLTFTPIPNAMMAGSDIFNGNSDRDTIVTNDFSANIVSRFIRFEPVTW 161
KT351732    WVTSFRVLSSLDGLTFTPIPNAMMAGSDIFNGNSDRDTIVTNDFSANIVSRFIRFEPVTW 161
W4ZF96      WVTSFRVLYSIDGLTWTAVQNSIMAGSNVFTGNDDRDTVVTNDFSSNIVARFIRIEPLTW 160
W4Z4Y0      WVTSYQISYSIDGKDWTLVKN-CDGGPKIFPGNSDGDSLIENGISPPVAARFIRLHPVTW 179

                1st FA58C             2nd FA58C
Q70JA0      ANHISMRFEVLGSIASKVEGPQKALGMKFGQIPDNAITASTEYDANHGAERSRLDTAAGA 221
KT351732    ANHISMRFEVLGSIASKVEGPQKALGMKFRQIPDNAITASTEYDANHGAERSRLDTAAGA 221
W4ZF96      TNHISMRFDILGTVASKIEGLQKALGMKDGQIPDSAITSSTQYDGNHGPERSRLDTVAGG 220
W4Z4Y0      YSHISLRWELIG--QGPVTCLSEKLGLEDYRIPDGAMTASTQYDSNTGPRRARLNLPVTG 237

Q70JA0      GKTGAWSARTNNVNQWLQVDLNSFYIITGVITQGRQDANQWVTGFRVESSDDGVTFNPIL 281
KT351732    GKTGAWSARTNDVNQWLQVDLNSFYIITGVITQGRQDANQWVTGFRVESSDDGVTFNPIL 281
W4ZF96      GRTGAWSARTNDVNQWLQVDLNSFYIITGVITQGRQDTDQWVTAFKVSSSDDGTNWNIIQ 280
W4Z4Y0      VLKGGWSALTLDQSQWLQVDLRGTYRVTGIITQGRADANEWVTSYNVAHSLNGINFNIIQ 297

Q70JA0      DCSGNQQ----------VFTGNADRNTKVTTEFARPITGRFLRIRPSSWNGHISMRLEIL 331
KT351732    DCSGNQQ----------VFTGNADRNTKVTTEFARPITGRFLRIRPSSWNGHISMRLEIL 331
W4ZF96      ACDGNDK----------VFSGNTDRNSKVTNTFDRPIVGRFLRIHPTTWNNHISMRLEIL 330
W4Z4Y0      IAASQQKKYSSYLTFLRAFTGNSDRTTQVTNYFSPPLTTRFIRVLPTMWFGHISLRMELL 357

    2nd FA58C          3rd FA58C
Q70JA0      GKGAVAGQRFEPLKLGMEDGRIADSQLSSSTCYDVNHCVDRARLNQVAGGGRTGAWSAQV 391
KT351732    GKGAVAGQRFEPLKLGMEDGRIADSQLSSSTCYDVNHCVDRARLNQVAGGGRTGAWSAQV 391
W4ZF96      GKGSVASNRFEPMKLGMEDGQIADSQLSSSTCYDSNHCVDRARLNQVAG----------- 379
W4Z4Y0      GAGPVAAILKDHVPLGLESNVIPDSSLTASSEFNADQGAKRGRLNLARVGSLRGAWSALV 417

Q70JA0      NDHSQWIEVDLLTDFMFSGVVIQGRSDANQWVTGYSLQYRPDGQ--TTLIDIVDENGAAQ 449
KT351732    NDHSQWIEVDLLTDFMFSGVVIQGRSDTNQWVTGYSLQYRPDGQ--TTLIDIVDENGAAQ 449
W4ZF96      ---------------MVINMTIT------------------------------------- 387
W4Z4Y0      NNANQWIQVDLLDPYRIISVATQGRQDESQWITSYKLACSTDGTTFDTVQGICTNPGADR 477
W4XXL8      ------------------------------WAPGLALKAETI------------------ 256

                                             3rd FA58C        4th FA58C
Q70JA0      IFSGSSDRDSLVISMLPLPVTARYVRIHPETWSGHISMRFELLGDGPINVVSTPGKLGIE 509
KT351732    IFSGSSDRDSLVISMLPLPVTARYVRIHPETWSGHISMRFELLGDGPINVVSTPGKLGIE 509
W4ZF96      ------------------------------------------------------------
W4Z4Y0      IFTGNVDRNTIVTSTLPVPQVCRYVRLMPVSWSGQISLRMEIYGEGPLTDQVR------- 530

Q70JA0      DGRVAASSLSASSCYDGNHCVDRSRLNQPRASPFTGAWSARTNDLDQWIQVDLRQAFEVT 569
```

```
KT351732   DGRIAASSLSASSCYDGNHCVDRSRLNQPRASPFTGAWSARTNDLDQWIQVDLRQAFEVT 569
W4ZF96     ------------------------------------------------------------
W4Z4Y0     --------------------------------VFCGDTCPRVHHPSGKVVQGMVDQVRVV 558

Q70JA0     GIITQGRNGWPHGQWVQSYQMSYSIDGKDWILVKGCLAETQIFPGNFDADSLVENAISPP 629
KT351732   GIITQGRNSWPNGQWVQSYQMSYSIDGKDWILVKGCLAETQIFPGNFDADSLVENAISPP 629
W4ZF96     ------------------------------------------------------------
W4Z4Y0     CVDTCPRVHCPSG-----------------YVVQGMVDQVRVFCG--DTCPRVHHPSGKV 599

                                      4th FA58C      5th FA58C
Q70JA0     VTARFFRLHPVRWNNHISLRWELIGMGPTTLAGSSRKLGLEDYRIPDSAITASTQFDANH 689
KT351732   VTARFFRLHPVHWNNHISLRWELIGMGPTTLAGSSRKLGLEDYRIPDSAITASTQYDANH 689
W4ZF96     ------------------------------------------------------------
W4Z4Y0     VQG-------------------MVDQVGVFCGDSCPRLHHPSGKVVQGMVDQVKVFCGDS 640

Q70JA0     GPQRARLNLPLSGALKGAWSALTLDHSQWLQVDLQGNYRVTGIITQGRASADQWVTMYKV 749
KT351732   GPQRARLNLPLSGALKGAWSALTLDHSQWLQVDLQGNYRVTGIITQGRASADQWVTMYKV 749
W4ZF96     ------------------------------------------------------------
W4Z4Y0     CPRVHHPSG--------------------------------------------------- 649

Q70JA0     AYSRNGEDFTTISSPGTPLQDKVFDGNSDRSTQVTNYFAPPFTARFIRVLPDEWHGHISM 809
KT351732   AYSRNGEDFTTISSPSTPLQDKVFVGNGDRSTQVTNYFAPPFTARFVRVLPDEWHGHISM 809
W4ZF96     ------------------------------------------------------------
W4Z4Y0     ------------------------------------------------------------

        5th FA58C          6th FA58C
Q70JA0     RIEILGAGPVANMLSDAVPLGLESTVIPDSSLTASSEWDPDHGAKRARLNLARVGILRGA 869
KT351732   RIEILGAGPVANMLSDAVPLGLESTVIQDSSLTASSEWDPDHGAKRARLNLARVGILRGA 869
W4ZF96     ------------------------------------------------------------
W4Z4Y0     ------------------------------------------------------------

Q70JA0     WSARTNNVNQWIQVDLLSPYRIFAVATQGRQDLNQWVTSYKIACSNDGATFDTVQGICTN 929
KT351732   WSAQTNNVNQWIQVDLLSPYRIFAVATQGRQDLNQWVTSYKIACSSDGATFDTVQGICTN 929
W4ZF96     ------------------------------------------------------------
W4Z4Y0     ------------------------------------------------------------

                                                     6th FA58C
Q70JA0     AAADRIFTANTDRNTIVTNSLPVPQVCRFVRLLPATWNSHISLRMELYGEGPLTE 983
KT351732   AAADRIFTANTDRNTIVTNSLPVPQVCRFVRLLPATWNSHISLRMELYGEGPLTE 984
W4ZF96     -------------------------------------------------------
W4Z4Y0     -------------------------------------------------------
```
